# Humans Process Dog and Human Facial Affect in Similar Ways

**DOI:** 10.1371/journal.pone.0074591

**Published:** 2013-09-04

**Authors:** Annett Schirmer, Cui Shan Seow, Trevor B. Penney

**Affiliations:** 1 Department of Psychology, National University of Singapore, Singapore, Singapore; 2 Duke/NUS Graduate Medical School, Singapore, Singapore; 3 LSI Neurobiology/Ageing Programme, National University of Singapore, Singapore, Singapore; Ghent University, Belgium

## Abstract

Humans share aspects of their facial affect with other species such as dogs. Here we asked whether untrained human observers with and without dog experience are sensitive to these aspects and recognize dog affect with better-than-chance accuracy. Additionally, we explored similarities in the way observers process dog and human expressions. The stimulus material comprised naturalistic facial expressions of pet dogs and human infants obtained through positive (i.e., play) and negative (i.e., social isolation) provocation. Affect recognition was assessed explicitly in a rating task using full face images and images cropped to reveal the eye region only. Additionally, affect recognition was assessed implicitly in a lexical decision task using full faces as primes and emotional words and pseudowords as targets. We found that untrained human observers rated full face dog expressions from the positive and negative condition more accurately than would be expected by chance. Although dog experience was unnecessary for this effect, it significantly facilitated performance. Additionally, we observed a range of similarities between human and dog face processing. First, the facial expressions of both species facilitated lexical decisions to affectively congruous target words suggesting that their processing was equally automatic. Second, both dog and human negative expressions were recognized from both full and cropped faces. Third, female observers were more sensitive to affective information than were male observers and this difference was comparable for dog and human expressions. Together, these results extend existing work on cross-species similarities in facial emotions and provide evidence that these similarities are naturally exploited when humans interact with dogs.

## Introduction

Humans are experts at reading another person’s facial emotions. They can do this fairly automatically and based on minimal cues obtained through only a casual glance. This is possible because all humans are comparable in the way they express emotions. Across different geographic and ethnic boundaries, shared facial movements exist that are linked to specific emotions, thus enabling the emergence of prototypes or templates that guide emotion recognition [1]. Notably, some of the facial movements that are shared among humans have equivalents in non-human animals, raising the possibility of cross-species recognition and communication. Here, we probed this possibility and assessed humans’ sensitivity to the facial behavior of dogs.

Our interest in the facial behavior of dogs derived from two research lines. First, there is growing evidence highlighting the dog’s ability to recognize and interpret human communication signals. Among others, the evidence includes the dog’s capacity to learn spoken words and symbols [2], to know when a human communication is intended for it [3], and to understand human gestures like pointing [4]. Importantly for the present study, there is also evidence that dogs are sensitive to human emotional expressions and use these expressions to guide their actions [5], [6]. For example, when faced with a novel object, dogs will look at their owner and more readily approach the object if their owner talks in an encouraging as compared to a discouraging manner [6]. Although this response may be learned, it could also be innate and derive from the presence of nonverbal cues that are shared between humans and dogs and that enable cross-species communication.

A second line of inquiry relevant for the present study is observational work in non-human animals suggesting evolutionary continuity in facial expressions. This work originated with Darwin’s theory of evolution and his attempts to describe and classify emotional behaviors across many species [7]. He, famously, concluded that some human expressions, such as the display of teeth in anger, have precursors in non-human primates and other mammals such as canids. Modern empirical work supports this view. Apes show a range of expressions, some of which parallel human emotion expressions [8]–[11]. For example, similar to the human smile or laughter, the ape play-face comprises a relaxed open mouth and vertically retracted lips. It is produced during playful encounters or invitations to play. The ape whimper-face, like the human sad face, comprises a protruding lower lip, raised eye brows and, occasionally, a forehead with horizontal wrinkles. Apes produce this expression in seemingly distressing situations or when they are pleading for the favor of others.

At present, only few attempts have been made to test Darwin’s proposition beyond primates. Some of these attempts were directed at the dog’s vocal repertoire, whereas others explored the dog’s postural and facial expressions. Focusing on vocalizations, Pongrácz and colleagues recorded dog barking across five different situations that presumably differed in their emotional meaning for the dogs [12]. These situations included the approach of a stranger, their handler prompting them to aggress, their handler preparing to go for a walk, being left alone, being shown a toy and being played with. Naive participants were given these situation labels and asked to classify dog barks accordingly. They could do this better than expected by chance. Additionally, they attributed different emotions to barks from different situations.

In the context of postural and facial expressions, researchers primarily relied on an observational approach whereby the researchers themselves classified and interpreted canid expressions. This approach led to the identification of a canid facial display that resembles the human happy face and the ape play face. It comprises an extended jaw and retracted lips and is produced during playful interactions [13], [14]. It has, thus, been linked to a joyful or happy state in the animal. Apart from the play-face, other canid expressions have been documented [13]–[15]. However, these have typically been studied in the context of specific situations and linked to dominance and submission rather than to emotions. For example, Fox (1970) placed canids in an observational area alone or together with a range of objects (e.g., a cloth) or other individuals and then reported ensuing expressions such as the agonistic jaw gap or exaggerated looking away. These expressions varied depending on the individual and the dynamics of the situation and were described with reference to an underlying behavior instead of an emotion.

To the best of our knowledge, there are only two published studies that explored canid facial emotion expressions experimentally. A study by Horowitz focused on the so-called “guilty look” of dogs. Dogs were presented with a forbidden treat in the absence of their owners. The owners, in the absence of their dogs, were rightly or wrongly told that their dog was “guilty” or that she behaved well. Based on this information, owners then either scolded or greeted their dog when reunited. For each condition, Horowitz then counted the frequency of facial displays that humans typically interpret as the “guilty look”. She found that dogs displayed the “guilty look” when scolded irrespective of whether the scolding was justified. Thus, she argued, the dogs’ facial expressions were more indicative of their owners’ behavior than their own behavior and may be unduly anthropomorphized.

A study that is particularly relevant for the present purpose was conducted by Bloom and Friedman [16]. In this study, one trained police dog was asked to sit and stay while his handler presented him with six different objects or spoken utterances aimed at eliciting happiness, sadness, surprise, disgust, anger, and fear. Ten photographs were made of the dog’s facial responses in each of these conditions and of his face during a 3-minute sit and stay period without additional stimulation. The latter served as a neutral control. The photographs were then ranked by three dog experts according to how successfully they conveyed the target emotion. Three of the most highly ranked photographs in each condition were then presented to a group of naïve participants, who rated them on six emotion dimensions. The ratings suggested some amount of cross-species emotion recognition. Specifically, photos from the happy condition were rated more strongly on the happy dimension than on any other dimension. Moreover, photos from the negative emotion conditions were rated more strongly on one or more of the negative emotion dimensions than they were on the happy dimension. Notably, however, some aspects of the rating results suggested limitations in cross-species communication. These included the fact that expressions from the neutral condition were rated just as happy as expressions from the happy condition and that participants misattributed several of the negative emotions.

The studies by Horowitz and by Bloom and Friedman significantly advance existing work on dog emotional expressions. They represent first attempts to assess dog faces using controlled lab-based procedures. Nevertheless, several methodological choices limit the conclusions that can be made. In the study by Horowitz, the lack of evidence for true guilt displays may be due to the choice of emotion. Guilt is a very complex emotion that emerges late during human development and that may be absent in dogs. As suggested by Bloom and Friedman, other emotions may well produce other results. In the study by Bloom and Friedman, the reliance on experts for selecting the face stimuli was problematic. It is unclear what criteria these experts used and to what extent they were influenced by their knowledge of human facial expressions. Another shortcoming is that expressions were generated by only one dog such that it is unclear whether they compare to expressions of other dogs and dog breeds. Thus, although Bloom and Friedman’s findings imply the possibility of cross-species facial emotion recognition, they cannot rule out anthropomorphizing and cannot be generalized to dogs at large.

The present paper presents an attempt to address these issues and to improve our understanding of dog facial expressions. Specifically, we sought to further probe whether these expressions are indeed recognized by humans and whether the underlying recognition processes compare to those recruited by human faces. To this end, we recorded the facial expressions of pet dogs and human infants under conditions that approximated what some believe to be universal antecedents of human happiness and sadness [17]–[20]. Still frames from the recordings were selected strictly based on situational criteria and concurrent non-facial behaviors (e.g., whimpering) that were previously linked to the target emotions. Nevertheless, because only one positive and one negative condition was used here, we henceforth refer to the isolated expressions as affective (i.e., positive/negative) rather than emotional (i.e., happy/sad). The expressions were presented to naïve human participants in two separate tasks assessing incidental/implicit and purposeful/explicit affect recognition, respectively. In the implicit task, dog and human expressions were used as primes in a lexical decision paradigm – a well established method for assessing automatic affective processes [21]. In this paradigm, facial expressions were followed by a letter string for which participants decided whether it was a proper English word. Words could be positive or negative such that implicit processing of facial emotions should facilitate lexical decisions to emotionally congruous as compared to incongruous words. In the explicit task, dog and human expressions were presented and participants rated each expression on an affect scale. In addition to presenting full face expressions, this task also used cropped expressions that exposed the eye region only. This was done to compare the facial parts that contribute to affect recognition in humans and dogs.

Based on existing work in dogs and the notion of evolutionary continuity in emotion or affect expression, we made the following predictions. First, we expected that naïve human observers, even without prior dog experience, would judge the target affect of dog faces more accurately than would be expected by chance. Second, we expected evidence for similarity in the processing of dog and human expressions. Such evidence could entail comparable explicit and implicit recognition results, comparable recognition from full and cropped faces, and comparable inter-individual differences.

Relevant for the latter point are previous studies that highlight a role of cultural exposure and biological sex in human emotion recognition. Among others, it was demonstrated that individuals judge the facial emotions of a familiar cultural group more accurately than the facial emotions of an unfamiliar group [22]. Additionally it was shown that, compared with men, women are more sensitive to task-irrelevant nonverbal emotional signals [23], [23,24] and that this sex difference has biological underpinnings involving sex hormones such as estrogen [23], [25]. Thus, if the processing of dog and human expressions is similar, these interindividual effects should extend to dog faces. Specifically, human observers should be less accurate for dog as compared to humans faces but this species effect should be smaller in dog owners relative to individuals who never owned a dog. Additionally, women should be more likely than men to show implicit emotion recognition of both dog and human faces.

## Methods

### Ethics Statement

This research was approved by the National University of Singapore Institutional Review Board and the Institutional Animal Care & Use Committee. It conforms with relevant national and international guidelines. Informed consent was obtained from both dog owners whose dogs contributed to the stimulus material of this study and human research participants. Participants arriving at the lab, were briefed about the study and asked to provide written consent.

### Participants

Seventy-one participants were recruited for this study. Seven participants, who scored lower than three standard deviations from the group mean in the lexical decision task, were excluded from data analysis. Of the remaining participants, 16 were female and had never owned a dog, 16 were female and had owned or currently owned a dog, 16 were male and had never owned a dog, and 16 were male and had owned or currently owned a dog. The group mean ages were 20 (St.Dev. 1.2), 20 (St.Dev. 1.3), 22 (St.Dev. 1.4), and 23 (St.Dev. 2), respectively. Their participation was recognized with course credit for an undergraduate psychology class or S$10.

### Stimuli

Stimuli were constructed for the implicit and the explicit emotion recognition tasks. Both tasks included a set of dog and human infant images. The implicit task additionally included a set of letter strings. The nature of the images and letter strings is detailed below.

#### Images

Dog expressions were collected from 33 dogs (Table 1) that visited a large public dog run together with their owners. The dogs were videotaped using a Canon HF 10 High Definition video recorder. In the positive condition, a dog was presented with a piece of food or its favorite toy depending on whether the owner reported the dog to be food or play motivated. This condition derived from prior work demonstrating a link between reward, joy, and motivation [26]. The baseline image was selected from the period before the dog was exposed to the treat. The affective image was selected after the owner initiated a movement to deliver the treat, but before the dog received and consumed the treat. In the negative condition, the dog was placed in a crate located in a deserted area of the dog run. This condition was modeled on the finding that social separation produces sadness [20]. The crate was 106 L×71 W×79 H cm in size and appropriate for all the dogs used in this study (i.e., they could stand and move around in it comfortably). The baseline image was taken while the dog owner was still visible to the dog. The affective image was taken after the owner had left and while the dog was showing known signs of distress (i.e., whining/pawing, licking, heavy panting). The dog was left alone in the crate for 5 minutes only. The conditions were presented in random order and were separated by five or more minutes during which the dog was freely moving around the dog run.

**Table 1:** Dog breeds.

Dog Breed	Frequency
Beagle	2
Boxer	1
Cocker Spaniel	2
Collie	1
Mixed breeds	9
Golden Retriever	2
Husky	1
Jack Russell Terrier	2
Japanese Spitz	1
Labrador Retriever	2
Maltese	2
Miniature Schnauzer	2
Shetland Sheepdog	2
Shiba Inu	1
Welsh Corgi	2
West Highland Terrier	1

Image capture was constrained by the dog’s position relative to the camera. For some dogs, movement compromised the recording angle for one or more conditions (e.g., they moved out of the camera frame or turned their head away) and these conditions or dogs had to be excluded from the study. Of the 33 recorded dogs, 24 were retained for each emotion condition.

**Figure 1:**
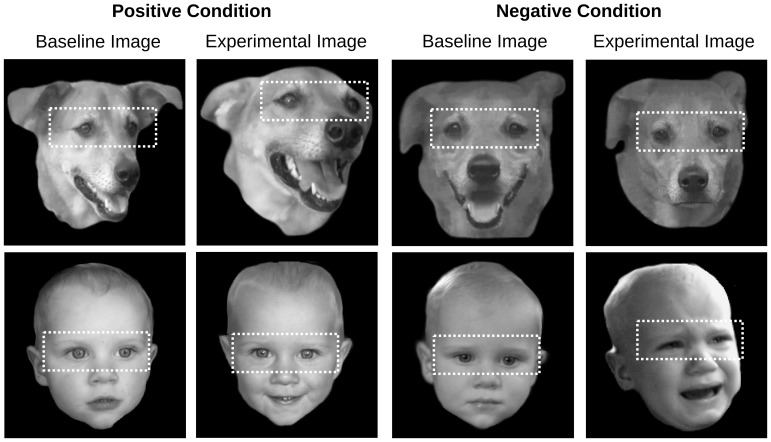
Exemplary images for the positive and negative conditions. The human infant in the figure was not used in the actual study. His guardians gave written informed consent, as outlined in the PLOS consent form, to publication of his photographs.

Images in the negative condition were degraded by the dog crate–which affected illumination and placed a grill in front of the dog’s face. Therefore, these images were edited using Adobe Photoshop to remove the grill and improve contrast and illumination. Additionally, all images were turned to gray-scale, the heads were isolated and the image edges were blurred. From these images we generated a second set of cropped images that maintained only the eye region including the eyebrows (Figure 1).

Human expressions were obtained in a similar fashion as the dog expressions. Because human adults may be expected to fail to respond to mild emotional provocation in a lab context, we opted to record the expressions of human infants. To this end, we invited 28 parents and their six to 12 month old infants into the lab and positioned the infant in a highchair facing a video camera. For the positive condition, infants were presented with a toy that was animated by the parent or the experimenter. For the negative condition, the parents were asked to leave the room such that the infant remained alone with the experimenter. If that did not distress the child, the experimenter hid from the child within the room. The maximal time of separation from the parents and the experimenter was five minutes. The child was reunited with his or her parents immediately after this period or after showing signs of distress.

Image capture was relatively less constrained for the infants than for the dogs because the infants could not move around freely. Nevertheless, not all infants responded to the emotional provocation such that we retained only 24 of the 28 infants for each emotion condition. As for the dog expressions, human expressions were isolated as still images from recordings taken prior to the affective provocation and during the affective provocation. The former served as baseline images and the latter served as experimental images. All selected images were turned to gray-scale, the head was isolated, and the image edges were blurred. From these images we generated a second set of cropped images that maintained only the eye region including the eyebrows.

While the explicit task used all the images resulting from these procedures, the implicit task used the experimental full face images only (i.e., baseline images and cropped images were excluded).

#### Letter strings

For the implicit task, the selected images were complemented by a set of linguistic stimuli consisting of 96 words and 96 pseudowords. The words were taken from the Affective Norms for English Words (ANEW) database [27]. They were selected based on their valence and depending on whether their lexical characteristics (i.e., word length, log frequency, number of morphemes, phonemes, and syllables, and orthographic neighborhood) were available in the English Lexicon Project [28]. The set comprised 48 positive words and 48 negative words equated for valence, arousal and the above-mentioned linguistic variables (Table 2). Pseudowords were constructed using the multilingual pseudoword generator Wuggy [29]. They were matched pairwise with the words in terms of length, orthographic neighbors, and number of syllables.

**Table 2:** Lexical and Sublexical Characteristics of Linguistic Stimuli.

	Negative Condition	PositiveCondition
**Valence**	2.36 (.60)	7.05 (.54)
**Arousal**	5.32 (.83)	5.16 (1.07)
**Log HAL frequency**	8.40 (1.38)	8.86 (1.88)
**Orthographic neighborhood**	2.10 (3.70)	1.58 (2.18)
**Number of letters**	6.44 (1.73)	6.46 (1.96)
**Number of syllables**	2.08 (.82)	2.00 (.83)
**Number of morphemes**	1.54 (.68)	1.40 (.71)

#### Paradigm

Participants, after arriving at the lab and providing informed consent, completed a short questionnaire that recorded their age, sex, whether or not they had owned a dog and how often they had interacted (e.g., petted, played) with a dog. The latter responses were solicited in categories of (i) 0 times, (ii) more than 0 times, (iii) more than 10 times and (iv) 30 or more times. Following this, participants were seated in front of a computer and performed an implicit and an explicit affect recognition task.

In the implicit task, participants saw images followed by letter strings and decided whether a given letter string formed a proper English word. A trial started with a white fixation cross presented on a black background for a duration of 400 ms. This was followed by a face image for 500 ms and a black screen for 200 ms. Then a letter string appeared for 300 ms and was replaced by another fixation cross that remained until participants responded or 3000 ms elapsed. Participants responded by pressing one of two buttons aligned horizontally on a keyboard using their right and left hands. The button assignment to word and pseudoword responses was counterbalanced across participants. The task started with 10 practice trials using stimuli not selected for the experimental trials. The remainder of the task was divided into two blocks with dog and human faces, respectively. Each block comprised 192 trials with 96 word and 96 pseudoword targets. Because we had only 48 primes for a given species or block (24 positive, 24 negative), primes were presented four times each and the presentation of primes within a block was not fully randomized. Instead, the repetition of primes across positive words, negative words, and their corresponding pseudowords was arranged to fall into the four quarters of each block in a manner counterbalanced across participants. The order of dog and human face blocks was counterbalanced also.

In the explicit task, participants were presented with the positive and negative experimental and baseline images and rated each image on a 5-point scale ranging from −2 (very sad) to +2 (very happy). A trial started with a white fixation cross presented on a black background for a duration of 400 ms. This was followed by the presentation of a human or dog expression in screen center and the aforementioned 5-point rating scale below the expression. Participants used the computer mouse to click on the relevant rating number. Image and scale remained until participants made their response. The next trial started after 500 ms. The explicit task comprised two blocks of 96 trials each. In the first block, participants saw only the eye region of each expression, whereas in the second block, they saw the full faces. Block order was not counterbalanced because the images were identical in the cropped and the full face condition with the exception of how much of the image was revealed. Moreover, we were concerned that viewing the full faces first would bias the rating of cropped faces. The presentation of images in each block was randomized.

In order to maintain the implicit nature of the implicit affect task, we decided to always present that task prior to the explicit affect task.

## Results

### Implicit Task

Reaction time and accuracy data from the implicit task were subjected to two separate ANOVAs with Prime (positive, negative), Target (positive, negative), and Species (human, dog) as repeated measures factors and Sex (male, female) and Dog Ownership (dog owner, non-dog owner) as between subjects factors. The predicted influence of prime affect on target responses should produce a significant Prime x Target interaction. Following positive primes, positive target words should be responded to faster and more accurately than negative target words. Following negative primes, the opposite pattern should emerge. Given these predictions, we only explored effects involving both Prime and Target.

#### Reaction times

**Figure 2:**
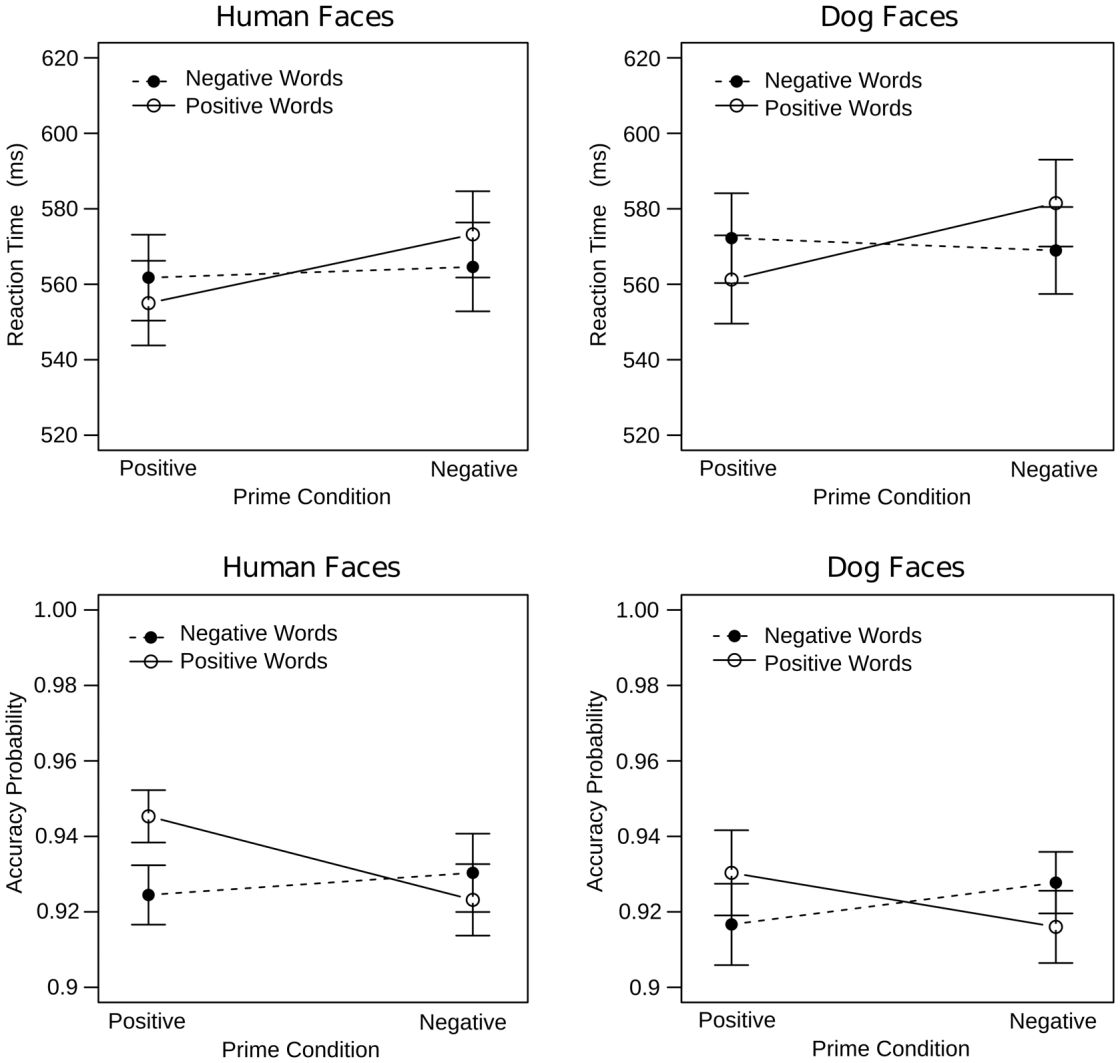
Accuracy and reaction time data from the implicit task. Both human and dog faces elicited significant priming in accuracy and reaction times. Target words were responded to faster and more accurately when they were affectively congruous, as compared to incongruous, with the preceding prime.

**Figure 3:**
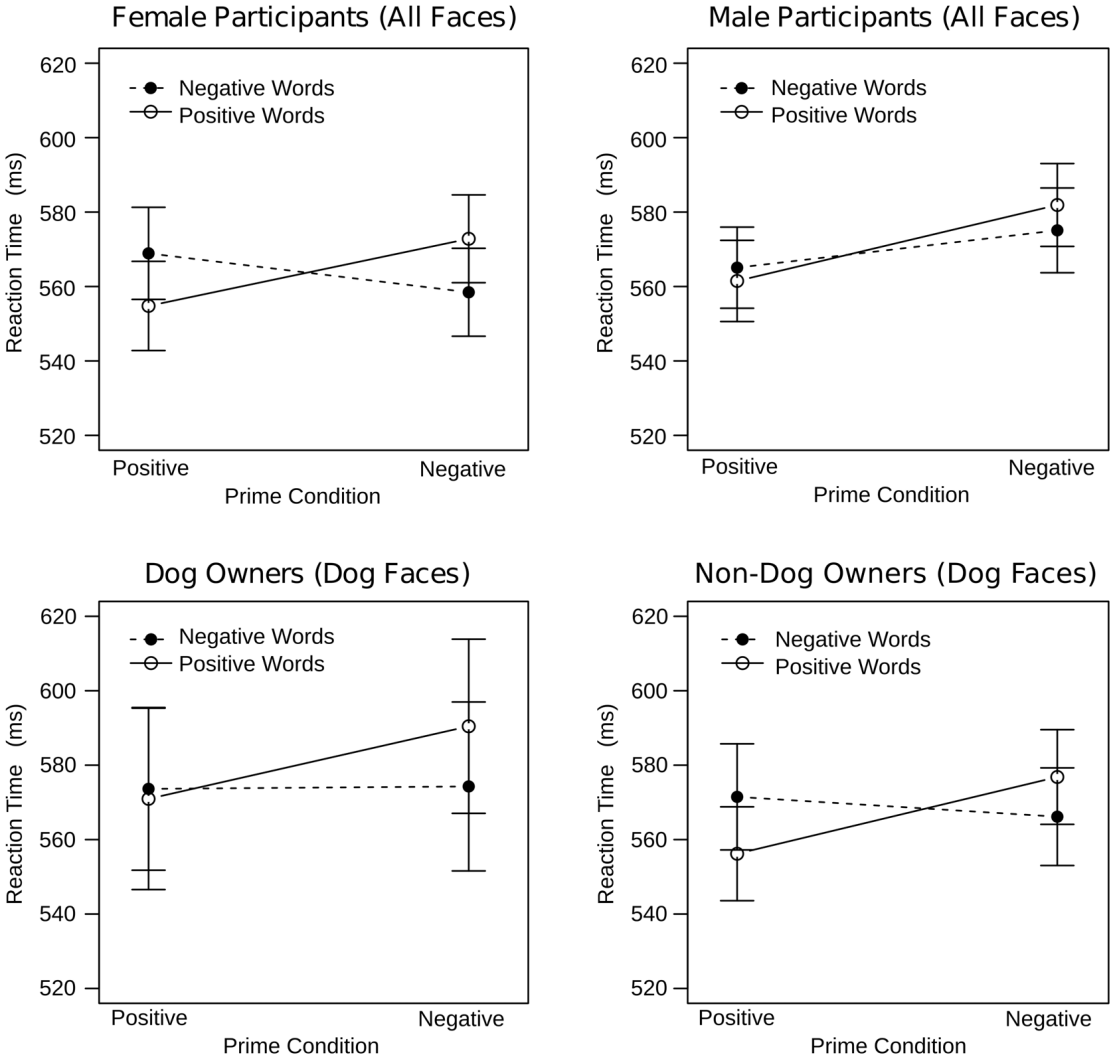
Interindividual differences in the reaction times of the implicit task. For both human and dog faces, women, but not men, showed significant priming (top row). For dog faces only, non-dog owners, but not dog owners, showed significant priming (bottom row).

Visual inspection of the reaction time data suggested a comparable priming effect for human and dog faces (Figure 2). Statistical analysis confirmed this impression and revealed a significant Prime x Target interaction (F(1,60) = 25.5, p<.0001) that was qualified by a three-way interaction including Sex (F(1,60) = 5.5, p<.05), but not Species (p>.3). The Prime x Target x Species x Dog Ownership interaction merely approached significance (F(1,60) = 3.4, p = .07). An exploration of the Prime x Target interaction in men was significant (F(1,30) = 4.3, p<.05). However, follow-up comparisons were only marginally significant or non-significant for positive (F(1,30) = 3.7, p = .06) and negative primes (p>.2), respectively. An exploration of the Prime x Target interaction in women was also significant (F(1,30) = 23.8, p<.0001; Figure 3). Additionally, women responded faster to positive than to negative words following positive primes (F(1,30) = 9.3, p<.01) and they responded faster to negative than to positive words following negative primes (F(1,30) = 13.3, p<.001).

Although the Prime x Target x Sex x Species interaction was non-significant (p>.4), we further explored the Prime x Target interaction in women, by examining dog faces only. This was done here and elsewhere in this study as an additional test to determine that affect recognition results were not simply driven by the human faces. We confirmed that this was not the case for the Prime x Target interaction found in women as dog faces from both the positive (F(1,30) = 6.8, p<.02) and negative (F(1,30) = 11.9, p<.001) condition significantly facilitated responses to affectively congruous words.

The Prime x Target x Species x Dog Ownership interaction was only marginally significant. Yet, we explored it further in an effort to determine whether Dog Ownership is critical for a robust priming effect with dog faces (Figure 3). We found the Prime x Target x Dog Ownership interaction to be significant for dog (F(1,60) = 4.4, p<.05), but not human faces (p>.6). For dog faces, non-dog owners showed a significant Prime x Target interaction (F(1,30) = 18.2, p<.001) indicating faster responses to positive than to negative words following primes in the positive condition (F(1,30) = 9.2, p<.01) and faster responses to negative than to positive words following primes in the negative condition (F(1,30) = 8.8, p<.01). Surprisingly, the same interaction was non-significant in dog owners (p>.17). These individuals merely showed a main effect of Prime (F(1,30) = 9.6, p<.01) indicating faster reactions times following primes in the positive as compared to the negative condition.

To determine whether dog experience, independently of dog ownership, is necessary for priming from dog faces to occur, we conducted a separate analysis for a subset of non-dog owners who indicated that they had never interacted with a dog (7 females, 9 males). This analysis corroborated the above results. An ANOVA with Prime, Target, and Species as repeated measures factors revealed a significant Prime x Target interaction (F(1,15) = 7.3, p<.05) that was unaffected by Species (p>.5). Follow-up comparisons demonstrated a significant Target effect for primes in the positive condition (F(1,15) = 6.4, p<.05) and a Target effect that approached significance for primes in the negative condition (F(1,15) = 3.6, p = .08).

#### Accuracy

Visual inspection of the accuracy data suggested a comparable priming effect for human and dog faces (Figure 2). Statistical analysis supported this impression with a significant Prime x Target interaction (F(1,60) = 8.8, p<.01) that was unqualified by Species (p>.8). The Prime x Target x Sex x Dog Ownership (F(1,60) = 5, p<.05) interaction was also significant. However, follow-up analyses indicated that neither men (F(1,30) = 3.1, p = .09) nor women (p>.1) showed a significant Prime x Target x Dog Ownership interaction. Hence, the Prime x Target interaction was followed up collapsed across groups revealing a significant Target effect for primes in the positive (F(1,60) = 13.4, p<.001), but not negative condition (p>.1).

Again we conducted two additional statistical tests that were, strictly speaking, not licensed by the overall analysis but that served as a means to probe the robustness of affect recognition from dog faces. Specifically, we tested whether the positive prime effect was present when dog faces were considered separately and found that this was not the case (p>.2). Additionally, we explored accuracy priming for individuals without dog experience and found that it was non-significant (p>.4).

### Explicit Task

**Figure 4:**
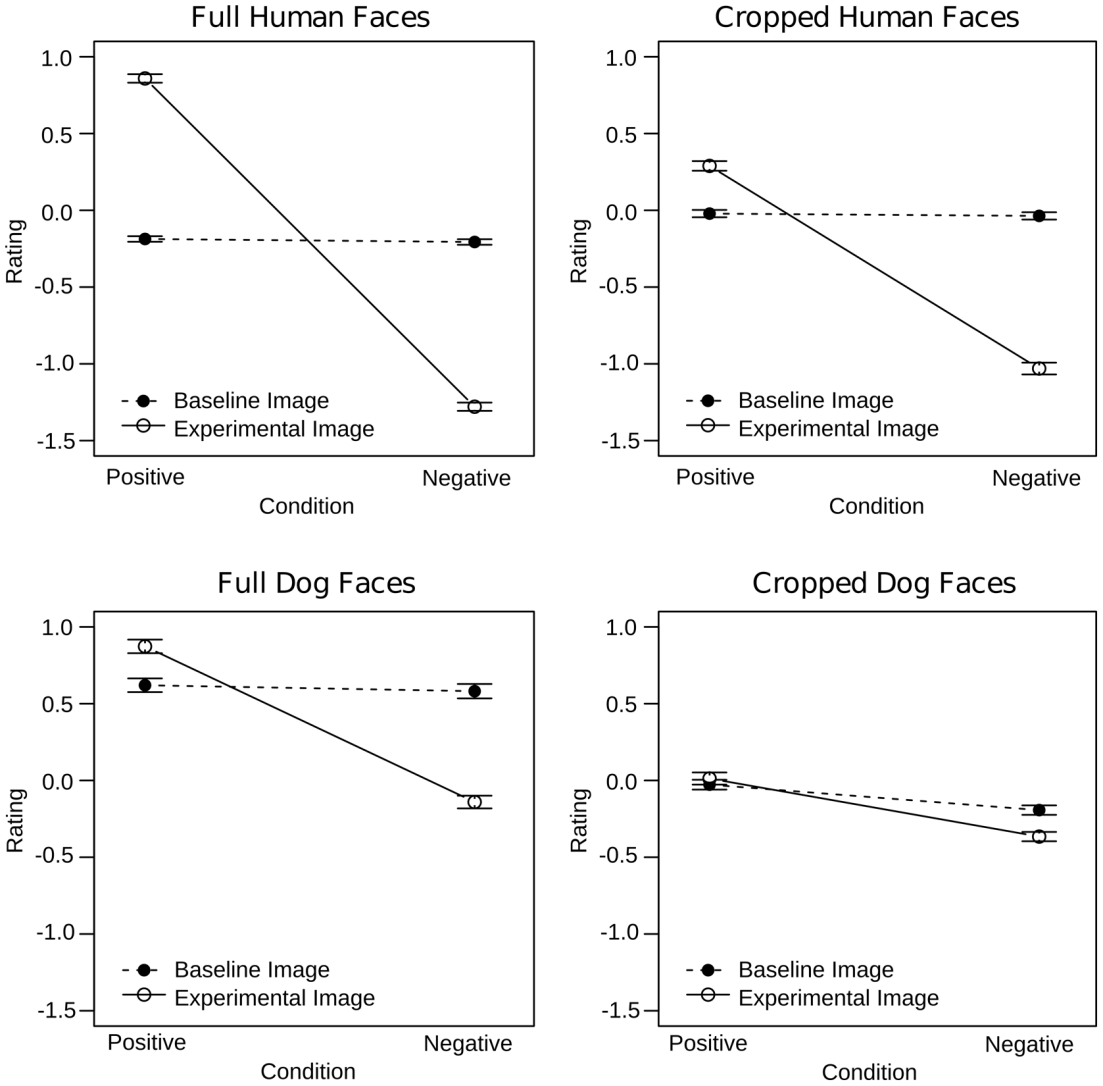
Rating results of the explicit task for full (left) and cropped (right) faces. Experimental images were rated as more positive and more negative than respective baseline images regardless of species. This effect was smaller in cropped than in full faces.

Visual inspection of the rating data suggested that affect was recognized successfully for both human and dog faces (Figure 4). To statistically probe these impressions, the rating data were subjected to an ANOVA with Condition (positive, negative), Image (experimental, baseline), Face (full, cropped), and Species (dog, human) as repeated measures factors and Sex (male, female) and Dog Ownership (dog owners, non-dog owners) as between subjects factors. Successful affect induction and recognition should be reflected by a Condition x Image interaction indicating that experimental images were rated more positively than baseline images for the positive condition and that they were rated more negatively than baseline images for the negative condition. Because the Condition x Image interaction was of primary interest, effects without both of these factors are not considered here.

As expected, the Condition x Image interaction was significant (F(1,60) = 1269.4, p<.0001). Additionally, Condition and Image interacted with Species (F(1,60) = 585, p<.0001), Face (F(1,60) = 385, p<.0001), and with Sex and Dog Ownership (F(1,60) = 9, p<.01). The Condition x Image x Species interaction could be explained by a stronger Condition x Image interaction effect for human (F(1,60) = 1808.6, p<.0001) as compared to dog expressions (F(1,60) = 228.7, p<.0001). Importantly, however, follow-up analyses of the Condition x Image interactions for each level of Condition were significant regardless of species. Human and dog experimental images were rated more positively as compared to baseline images in the positive condition (human: F(1,60) = 780.5, p<.0001; dog: F(1,60) = 41.5, p<.0001) and were rated more negatively as compared to the baseline images in the negative condition (human: F(1,60) = 1421.9, p<.0001; dog: F(1,60) = 338.6, p<.0001; Figure 4).

**Figure 5:**
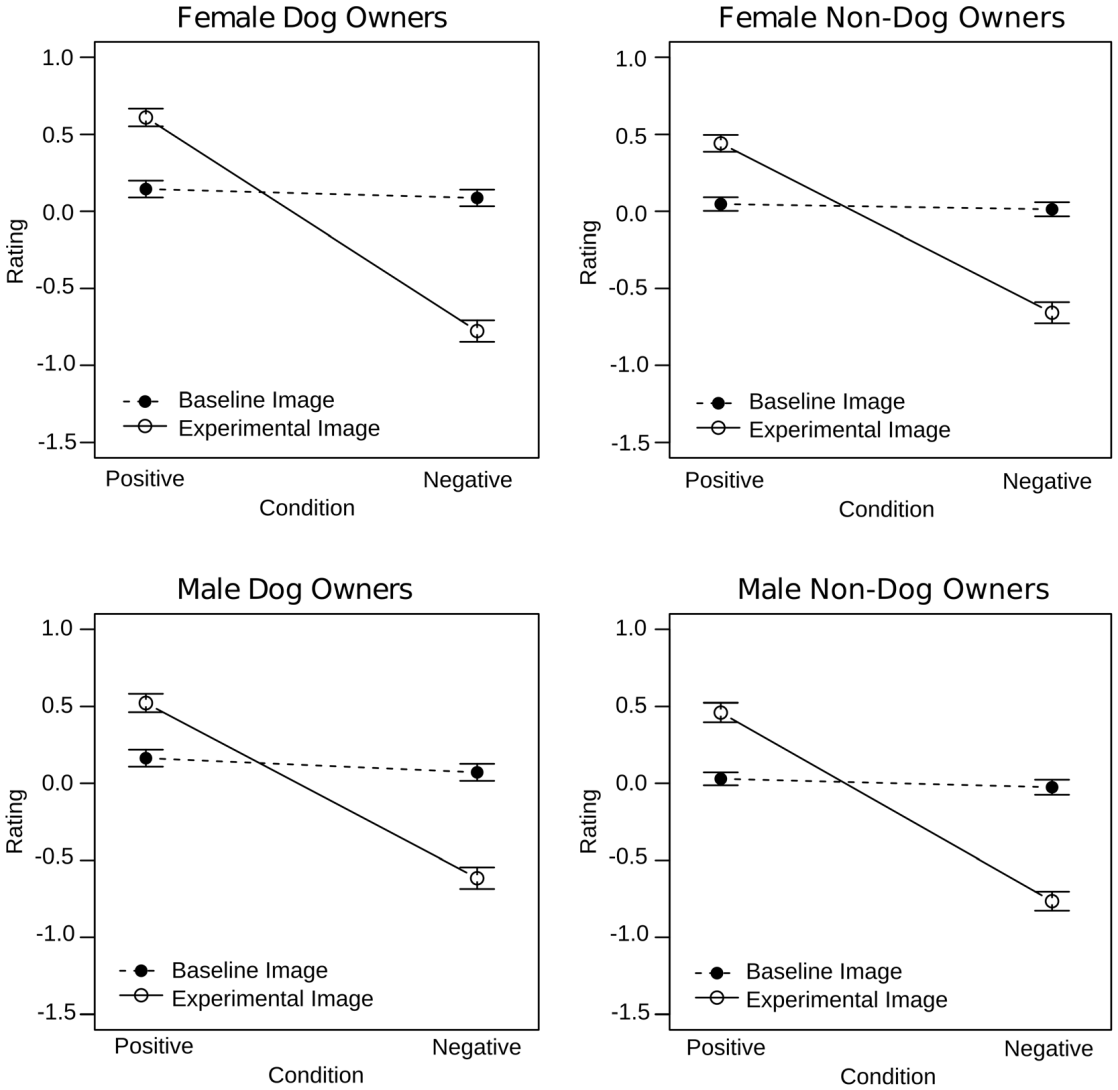
Interindividual differences in the rating results of the explicit task. Female dog owners were better at differentiating experimental from baseline i mages than female non-dog owners. There was no such difference between male dog and non-dog owners.

The Condition x Image x Face interaction was followed up for full and cropped faces separately. The Condition x Image interaction effect was greater for full (F(1,60) = 1493.5, p<.0001) as compared to cropped faces (F(1,60) = 443.3, p<.0001). In all cases, however, the Image effect was significant for the positive (full: F(1,60) = 883.8, p<.0001; cropped: F(1,60) = 56.7, p<.0001) and negative conditions (full: F(1,60) = 1164, p<.0001; cropped: F(1,60) = 555.9, p<.0001) indicating that affect was successfully recognized from both full and cropped faces (Figure 5). Again, a separate analysis was conducted to test whether the Condition x Image interaction observed for cropped faces was significant when only dog faces were considered. As this was the case (F(1,60) = 17.15, p<.001), we explored the Image effects for each level of Condition. In the negative (F(1,60) = 40.4, p<.0001), but not in the positive condition (p>.2), experimental images were rated as significantly more affective than baseline images.

Follow up of the Condition x Image x Sex x Dog Ownership interaction revealed a significant Condition x Image x Sex interaction in dog owners (F(1,30) = 643.4, p<.0001). Further exploration indicated that the Condition x Image interaction effect was larger in female (F(1,30) = 417.9, p<.0001) as compared to male dog owners (F(1,30) = 240.8, p<.0001), but was otherwise comparable. In non-dog owners, the Condition x Image x Sex interaction was non-significant (p>.1). Instead, the Condition x Image interaction was significant and ultimately comparable to that reported in dog owners (F(1,30) = 626.35, p<.0001; Figure 5).

A separate analysis was carried out in individuals who never owned a dog and additionally indicated that they had never once interacted with a dog (7 females, 9 males). Their data was subjected to an ANOVA with Condition, Image, Species, and Face as repeated measures factors. This analysis revealed comparable effects to those reported above. These effects included an interaction of Condition and Image (F(1,15) = 556.8, p<.0001) that was qualified by an interaction with Face (F(1,15) = 57.3, p<.0001) and Species (F(1,15) = 217.5, p<.0001). Importantly, following up the latter effect indicated that the Condition x Image interaction for dog faces was significant (F(1,15) = 43.6, p<.0001). In the positive condition, dog faces were rated more positively for experimental as compared to control images (F(1,15) = 4.5, p = .05), whereas in the negative condition, the opposite pattern emerged (F(1,15) = 62.5, p<.0001). The Condition x Image x Species x Face interaction was non-significant (p>.9).

The failure of dog ownership to significantly enhance dog affect recognition was surprising. Based on previous research showing cross-cultural differences in the recognition of human facial expressions, we expected familiarity with dogs to boost performance on dog trials and to decrease the Species effect. That this was not the case may have been due to participants in the non-dog owner group having had prior experiences with dogs. To test this possibility we explored the effect of self-reported dog experiences. Sixteen non-dog owners reported never having interacted with a dog, 9 reported having interacted more than 0 times, 6 reported having interacted more than 10 times, and 1 reported having interacted 30 or more times with a dog. These participants were dummy-coded as 0, 1, 2, and 3, respectively and their codes were used in a series of Pearson correlation analyses with the individual rating differences between the experimental and baseline images as the independent variable. Specifically, we collapsed across full and cropped faces and subtracted baseline ratings from experimental ratings in the positive condition and experimental ratings from baseline ratings in the negative condition. Significant results were obtained for dog faces from the positive (r = .34, p = .05, two-tailed) and negative condition (r = .43, p<.05, two-tailed) but not for human faces (ps>.36).

## Discussion

Despite much interest in the presence of human-like nonverbal expressions in other species, only a few attempts have been made to explore whether and how these expressions are accessible to untrained human observers. The present study represents such an attempt. It used naturalistic facial displays of dogs and human infants elicited under neutral conditions and emotional provocation. The displays were presented to naïve participants with the following two objectives. First, we hoped to determine whether naïve participants identify dog affect with better-than-chance accuracy. Second, we asked whether the mechanisms that allow such identification compare to those engaged for human affect.

### Can Humans Identify Facial Emotions in Dogs?

The first objective was addressed in an explicit affect recognition task in which participants rated the affect of dog and human infant expressions. Experimental images obtained during emotional provocation were rated differently from baseline images obtained prior to emotional provocation. In the positive condition, experimental images received more positive ratings, whereas in the negative condition they received more negative ratings than the baseline images. Notably, these rating effects were present not only for human, but also for dog expressions and did not presuppose prior experience with dogs. Hence, we conclude that dogs, like human infants, were sensitive to our emotional provocations and produced expressions of which aspects were recognizable to untrained human observers.

What aspects the human observers recognized, however, remains debatable. Although we used emotion specific provocations with the dogs, we cannot be certain that these emotions were actually elicited and visible in the dogs’ faces. It is possible that instead of provoking happiness or sadness, we elicited pleasant surprise or fear, respectively. Moreover, even if we elicited happiness and sadness, the facial changes may have simply reflected changes in affective tone rather than a specific emotion. Exploring only one positive and one negative emotion prevented us from rejecting these possibilities. Therefore, the present results provide insights only on the communication of positive and negative affect and leave the question of emotion specific dog facial expressions for future research.

Nevertheless, the finding that dogs produce facial expressions of which the underlying affect is accessible to humans without dog experience is interesting. It implies that our emotional provocations produced fairly consistent facial displays in the dogs. Moreover, the fact that these displays could be interpreted by individuals who had only their human emotion recognition experience to go by suggests cross-species similarities in affective communication. This similarity is in line with Darwin who traced human facial expressions to a mammalian ancestor. Additionally, it accords with extant work on dog emotional expression suggesting that humans can recognize certain vocal [12], [30] and facial cues [13], [14], [16]. Notably, our findings extend the work on facial cues by addressing previous methodological shortcomings and by providing experimental evidence that humans discriminate both negative and positive from neutral dog faces.

### Do Humans Process Human and Dog Faces in Similar Ways?

The second objective of this study concerned possible similarities in the processing of human and dog expressions and was tackled by exploring processing automaticity, critical facial features, and interindividual differences (i.e., sex difference, experience) that mark recognition success of human and dog affect.

#### Automaticity

The automaticity associated with recognizing human and dog expressions was explored in an implicit face processing task. Prior work revealed that human observers are sensitive to emotions in human faces even when these faces are task-irrelevant [23], [31]. Dedicated processing systems have been postulated that enable this effect and that rely largely on low spatial frequency information transmitted via a fast, magnocellular pathway from the retina to the thalamus and from there to the amygdala [32], [33]. Moreover, the amygdala has been construed as a relevance detector that responds to emotional human faces because it identifies their structural configuration as potentially important for the observer [34]. The present observation of comparable priming from human and dog faces raises the possibility that dog faces recruit similar automatic processes.

Other aspects of the present priming results concern sex differences and differences in the priming pattern for reaction time and accuracy data. Sex differences were observed in that reaction time priming from human and dog faces was significant in female participants only. This difference was predicted and its implications will be discussed below in the section on interindividual differences. Differences between reaction time and accuracy priming emerged in that reaction time priming was comparable for the positive and negative condition, whereas accuracy priming irrespective of sex, showed only for the positive condition and only for human faces.

That priming should be weaker for accuracy than for reaction times is unsurprising. The task was relatively easy and participants were asked to respond both quickly and accurately without sacrificing accuracy for response speed. Thus, participants performed close to ceiling leaving little room for accuracy effects to emerge. That such effects were nevertheless evident for the human positive condition suggests that positive faces are a fairly powerful prime. Moreover, the absence of accuracy effects for human negative faces, dog positive faces and dog negative faces suggests that these primes were less powerful. This may be due to the relatively higher frequency with which we encounter human positive as compared to the other faces. After all, positive faces form a major component of human greeting rituals and other interactions even if they are not always genuine. Being more exposed to human positive as compared to other faces can hence be expected to create processing differences such that human positive faces become more accessible and more likely to impact other concurrent mental processes.

#### Critical facial features

To test whether human and dog face processing involves a similar set of critical facial features, we explored the rating results for full and cropped images. Like the accuracy for human faces, the accuracy for dog faces was greater when faces were fully presented than when they were cropped and the drop in accuracy was largely comparable across species. Detailed analyses for cropped dog faces, indicated that experimental images were rated as more affectively valenced than baseline images for the negative, but not the positive condition. This suggests that for sadness or related negative states, but perhaps not happiness, facial features in the eye region are shared across species.

Why may such features be absent for happiness? To answer this question, we need to consider how emotions affect the eye regions in humans. In the case of human happiness, a “squinting” of the eyes has been noted that is caused by an activation of the orbicularis oculi muscle [35]. Although this squinting has long been considered the distinguishing feature of a Duchenne or true smile [36], recent research suggests that it is an unreliable marker of emotion and that it is possibly learned [37]. If true, one would not expect dogs to show a similar squinting despite the fact that like humans they have an orbicularis oculi muscle [38]. Moreover, this would explain why human participants in our study were unable to differentiate positive from neutral dog expressions when presented with the eye region only.

In the case of human sadness, a raising of the inner eyebrows is affected by contraction of the medial aspect of the frontalis muscle [35]. Again this muscle is present in dogs enabling similar brow movements [38]. However, because the present images were created as stimulus material, we have no measurements of facial muscle activity. We can, therefore, say little about the exact muscular involvement in the target expressions. Yet, that a very circumscribed region around the eyes enabled human participants to discriminate experimental from baseline images in the negative condition for both human infants and dogs points to species overlap in the facial features of emotion expression that possibly involve a raising of the inner brows.

#### Sex differences

If human and dog faces are processed in similar ways, then the sex differences normally elicited by human nonverbal expressions should also be elicited by the nonverbal expressions of dogs. Specifically, previous work on human faces and voices showed that women are more emotionally sensitive than men, especially in situations in which nonverbal expressions are task-irrelevant and thus processed implicitly. Moreover, some but not all sex differences disappear when emotions are assessed explicitly [39], [40]. Based on these findings, we expected women to be more sensitive than men to emotional aspects of human infant and dog expressions and for this sex effect to be more pronounced in the context of implicit as compared to explicit processing.

In line with these expectations, sex differences were substantial in the implicit task. Women, but not men, demonstrated affective priming for both dog and human faces in their reaction times. Women made faster lexical decisions to words that matched the primes’ affective valence as compared to words that did not match. In comparison, sex differences in the explicit task were less drastic. Both male and female participants were able to discriminate experimental from baseline images across affective conditions and species. Female participants simply showed greater discrimination than males. Somewhat surprisingly, however, this difference in discrimination was significant only among dog owners.

A heightened female sensitivity to human socio-emotional signals has been reported repeatedly and linked to both environmental influences and biological determinants. Environmental influences presumably arise from parental modeling and the internalization of societal norms [41]. Biological determinants have been traced to the sex chromosomes [42] and to differences in the concentration of hormones and neuropeptides such as estrogen [25] and oxytocin [23], [43]. Although present in both men and women, these messengers play different functional roles in that they promote affiliative or pro-social tendencies particularly in women [43].

The present sex differences likely arise from a combination of environmental and biological factors. However, both probably contributed somewhat differently to performance in the implicit and explicit tasks. While in the implicit task, biological factors may have been more important than environmental factors, the opposite was likely true for the explicit task. This possibility is in line with prior research that has linked estrogen to the processing of task-irrelevant nonverbal signals [25]. Additionally, it accords with the fact that sex differences in the explicit task showed for dog owners only and thus likely arose from life experiences that come with caring for a dog. One hypothesis here would be that dog ownership increases the frequency of interactions with strangers and that this in turn increases social skills particularly in women who may already be more socially inclined.

#### Experience-based differences

Apart from sex differences, we were also interested in whether and how individual experiences with a particular species shape emotion recognition. Again, existing work on human faces found such experiences to be facilitative. Individuals are better at recognizing facial emotions from a familiar ethnic group as compared to a less familiar or unfamiliar group [22]. Thus, one would expect human observers to show greater emotion sensitivity to human as compared to dog faces and for this difference to be more pronounced in individuals with little as compared to substantial dog experiences.

In line with this, humans showed an own-species advantage in that they seemed more sensitive to human as compared dog facial affect. This is reminiscent of the own-race advantage for emotion recognition [22] and in line with the notion that familiarity and learning shape sensitivity to race or species typical emotion cues. Contrary to this notion, however, dog ownership failed to reduce the own-species advantage. In the implicit task, dog owners were less affected than non-dog owners by the emotional congruity between dog primes and target words. Instead, dog owners showed a prime main effect suggesting that they processed primes in an explicit, but not necessarily more effective way. In the explicit task, the effect of dog ownership showed only in an interaction with sex and irrespective of species. Analysis of this interaction revealed that female dog owners performed better than male dog owners giving rise to the possibility that the social exposure that comes with caring for a dog leads to a sex-specific improvements in social perception.

Together, the effects of dog ownership indicate that owning a dog changes the way humans engage with dog images, but fails to significantly enhance dog affect recognition. This somewhat puzzling result accords with prior research that found largely comparable performance in dog owners and novices for dog expression recognition [12], [16]. Their comparable performance may be explained by the fact that dogs are common among humans such that even non-dog owners have some amount of dog experience that informs their emotion judgments. In support of this, we found the frequency of dog interactions to predict emotion rating results. Non-dog owners were better able to discriminate between experimental and baseline images the more they had previously interacted with dogs. Thus, dog experience seems more relevant than dog ownership per se. Moreover, that the greater experience of dog owners does little to further enhance dog affect recognition may be because this experience is typically limited to one dog (i.e., their own pet) and may not help much in the acquisition of species-specific cues.

## Conclusions

In sum, the present study shows that humans recognize positive and negative affect in the facial behavior of dogs and that they can do so without having ever interacted with a dog. Additionally, a number of similarities were revealed between the processing of human and dog expressions. Naive human participants processed both types of expressions implicitly, recognized negative states from the eye region only, and demonstrated species independent sex differences. These findings are in line with existing reports that established expressive overlap between humans, dogs, and other canids and that points towards evolutionary continuity in the emergence of nonverbal communication [13], [14], [16], [30]. The present study extends these reports by showing that untrained observers can leverage on this overlap, thereby suggesting that it forms the basis for successful human-dog interactions.
